# Mental health outcomes and associated factors among vaccinated and unvaccinated teachers against COVID-19 infection in Bangladesh

**DOI:** 10.3389/fpsyt.2022.896419

**Published:** 2022-08-03

**Authors:** Md. Dhedharul Alam, Asraful Islam, Md. Sanwar Hossain, Afsana Hossain, Delara Akhter, Md. Masum Haider, Yi Xu

**Affiliations:** ^1^Department of Psychiatry, The First Affiliated Hospital, Zhejiang University School of Medicine, Hangzhou, China; ^2^The Key Laboratory of Mental Disorder Management in Zhejiang Province, Hangzhou, China; ^3^Department of Psychology, Jagannath University, Dhaka, Bangladesh; ^4^Department of Statistics, Jagannath University, Dhaka, Bangladesh; ^5^Department of Plant Pathology, Bangabandhu Sheikh Mujibur Rahman Agricultural University, Gazipur, Bangladesh; ^6^Department of Genetics and Plant Breeding, Sylhet Agricultural University, Sylhet, Bangladesh; ^7^Department of Physics, Mawlana Bhashani Science and Technology University, Tangail, Bangladesh

**Keywords:** Bangladesh, COVID-19, teachers, immunization, mental health outcomes, refusal, uptake

## Abstract

**Background:**

Vaccination of teachers is recommended during the COVID-19 pandemic to reduce the risk of infection for themselves and their students, as well as to encourage their parents to get immunized. The present study investigated the mental health outcomes and associated factors among vaccinated and unvaccinated teachers against COVID-19 infection in Bangladesh.

**Methods:**

A cross-sectional survey was conducted in Bangladesh from March 4 to September 9, 2021. The frequency of symptoms of psychological distress, depression, anxiety, stress, post-traumatic stress disorder (PTSD), insomnia, and fear was assessed using the Bangla versions of the GHQ-12, PHQ-2, GAD-2, PSS-4, PC-PTSD-5, ISI, and FCV-19S scales, respectively.

**Results:**

A total of 1,527 Bangladeshi teachers completed the questionnaire, with 678 (44.4%) being vaccinated and 849 (55.6%) being unvaccinated. Compared with unvaccinated teachers, vaccinated teachers had a statistically significant lower prevalence of psychological distress (35.8 vs. 42.9%), depression (37.6 vs. 46.4%), anxiety (31.9 vs. 45.1%), stress (18.3 vs. 32.0%), PTSD (33.0 vs. 43.8%), insomnia (25.2 vs. 36.9%), and fear symptoms (23.3 vs. 29.6%). Among vaccinated teachers, participants with master’s or lower degree levels had significantly higher symptoms of depression, stress, and fear than other education levels. Respondents with children had a significantly higher risk of depression, anxiety, stress, and fear symptoms than those who did not have children. Participants who lost family members, friends, or colleagues due to the COVID-19 pandemic had a significantly higher chance of experiencing symptoms of anxiety, PTSD, and fear than those who did not. On the other hand, unvaccinated male teachers were significantly associated with a higher risk of all mental health outcomes except psychological distress and PTSD symptoms compared to female teachers. Participants who were smokers had a significantly higher chance of anxiety, stress, and fear symptoms than non-smokers. Compared to participants with strong social support, those with poor social support had a higher risk of all mental health outcomes except PTSD symptoms.

**Conclusion:**

This study suggests emphasizing the vaccinated to unvaccinated teachers as soon as possible to control the infection and improve mental health outcomes. Vulnerable teachers also required special attention, health-related education, and psychological support.

## Introduction

The globe is currently in the grip of a Coronavirus Disease 2019 (COVID-19) pandemic. Since the commencement of the COVID-19 pandemic in 2019, around 225 countries and 227 million people have been afflicted with the virus, which has killed about 4.6 million people (as of September 19, 2021) (1). The COVID-19 pandemic affects people from all walks of life and all sectors of society. All educational institutions have been closed, and teachers and students have been forced out of the classrooms (2). As of mid-April 2020, school closures had touched 1.5 billion children and youth in 195 countries, from pre-primary to university level (3). Emerging research indicates the COVID-19 outbreak had a severe impact on teachers. Teachers were concerned about their students’ physical and mental wellbeing and their own (4). According to research conducted before the outbreak, many researchers have stated that teachers are at significant risk of experiencing depression, anxiety, stress, and burnout as a result of being exposed to a wide range of professional stressors in their everyday duties (5, 6).

However, the COVID-19 problem has exacerbated the situation (7). A systematic study of COVID-19’s impact found that teacher depression, anxiety, and stress levels were much higher than pre-COVID-19 levels (8). An extensive cross-sectional survey among 18,521 teachers in China between February 21 and February 29, 2020, during the COVID-19 outbreak, found that sleep disturbance (35.5%), somatic discomfort (25.3%), anxiety (17.7%), depression (4.0%), self-injury or suicidal thoughts (2.8%) (9). Moreover, Ozamiz-Etxebarria et al. (10) conducted a meta-analysis of eight studies published from December 1, 2019, to June 15, 2021, reporting that teachers experienced high rates of depression (19%), anxiety (17%), stress (30%) during the COVID-19 pandemic. Additionally, during the epidemic, studies of teachers found an increase in post-traumatic stress disorder (PTSD), distress, fear, workplace loneliness, burnout, fatigue, and poor quality of life (11–14).

Bangladesh, where the current study was done, is a South Asian country where COVID-19 has significantly impacted its education system (15). The first COVID-19 case was reported in Bangladesh on March 8, 2020, and as of September 19, 2021, the country had 1.5 million verified COVID-19 cases and 27,225 deaths (Supplementary Figures 1, 2) (16). Bangladesh is among the top 28 countries contributing to 0.68% of the COVID-19 cases in the world. On March 17, 2020, Bangladesh’s government decided to keep all the educational institutions closed as a precautionary measure against coronavirus (17). The Bangladeshi government has announced the reopening of schools and colleges from September 12, 2021, after the closure of around 18 months (18). It will reopen the universities in the country from October 15, 2021 (19). Teachers were forced to switch from traditional face-to-face to online teaching with only a few days’ notice when all educational institutions abruptly closed. Unfortunately, like other countries, Bangladesh continues to face challenges such as a lack of technical and infrastructural infrastructure and teacher training (20, 21). Therefore, it affects teachers’ physical and mental health (22). Ferdous and Shifat (23) conducted a study among 46 English Language Teaching (ELT) teachers and English as Foreign Language (EFL) students from private universities in Bangladesh found that worsening mental health conditions were embraced both by ELT teachers and EFL learners during the COVID-19 outbreak.

Vaccines are one of the most effective strategies for preventing COVID-19 infection and its consequences and complications (24). The first COVID-19 human clinical trial began on March 3, 2020 (25). As of September 20, 2021, 577 million COVID-19 vaccination doses were delivered globally (1). Teachers are among several priority groups to be vaccinated under national vaccine rollout plans in 72% of countries (146 out of 204) worldwide (26). On January 27, 2021, Bangladesh began providing COVID-19 vaccines, with bulk vaccination starting on February 7, 2021, and the second dosage beginning on April 8, 2021 (27). The teachers and officials of educational institutions in the country started vaccines against COVID-19 by March 30, 2021 (28). As of August 12, 2021, 90% of university teachers had been immunized in Bangladesh (29). As of September 19, 2021, the number of first doses administered in Bangladesh exceeds 22.4 million, the number of second doses administered exceeds 14.8 million, and the total vaccination exceeds 37.2 million (Supplementary Figure 3) (30). Ideally, a high enough percentage of the population will be immunized, safeguarding those who are unimmunized, a process is known as “herd immunity.” It has been estimated between 55 and 82% of populations would need to be vaccinated to reach herd immunity for COVID-19, depending on varying biological, environmental, socio-behavioral factors, and infection rates within each country (31).

Given the considerable increase in anxiety and depression symptoms connected to the stress of the COVID-19 pandemic (32), immunization could alleviate anxiety and depressive symptoms. However, it is not known whether the psychological status would be affected after COVID-19 vaccination among teachers. One study conducted among 1,779 adults in Germany between January 1, 2021, to January 11, 2021, showed that COVID-19 vaccination could positively correlate with COVID-19-related anxiety and fears (33), while another study investigated among 34,041 general public in China between January 29 to April 26, 2021, found that psychological stress levels after getting vaccinated significantly decreased (34). In addition, a cross-sectional survey of 363 health care workers in Turkey indicated that COVID-19 vaccination was not linked to secondary traumatic stress, anxiety, and depression symptoms (35). As a result, it is critical to look into how this COVID-19 immunization affects mental health, particularly among teachers. However, there have been no studies on the mental health outcomes of COVID-19 vaccination on both vaccinated and unvaccinated teachers in Bangladesh yet. Therefore, we conducted a cross-sectional survey to assess the mental health outcomes and associated factors among vaccinated and unvaccinated teachers against SARS-CoV-2 infection in Bangladesh. This study looked into the prevalence of psychological distress, depression, anxiety, stress, PTSD, insomnia, and fear among vaccinated and unvaccinated teachers against SARS-CoV-2 infection in Bangladesh and explored its contributing factors.

This study had three goals based on these considerations. First, we sought to determine the prevalence of psychological distress, depression, anxiety, stress, PTSD, insomnia, and fear among vaccinated and unvaccinated teachers against SARS-CoV-2 infection in Bangladesh. Second, we sought to identify differences in the prevalence of psychological distress, depression, anxiety, stress, PTSD, insomnia, and fear symptoms among vaccinated and unvaccinated teachers in Bangladesh. Third, we sought to explore which socio-demographic factors could significantly predict mental health outcomes in the group of vaccinated and unvaccinated teachers against COVID-19 infection in Bangladesh. This study had three research questions. First, what is the prevalence of psychological distress, depression, anxiety, stress, PTSD, insomnia, and fear symptoms among vaccinated and unvaccinated teachers against SARS-CoV-2 infection in Bangladesh? Second, are there any differences in the prevalence of psychological distress, depression, anxiety, stress, PTSD, insomnia, and fear symptoms among vaccinated and unvaccinated teachers against SARS-CoV-2 infection in Bangladesh? Third, which socio-demographic factors do significantly predict mental health outcomes in the group of vaccinated and unvaccinated teachers against SARS-CoV-2 infection in Bangladesh? Based on these objectives and research questions, we hypothesized that vaccinated teachers had a lower prevalence of mental health outcomes against COVID-19 infection in Bangladesh than unvaccinated teachers. This research will add to our understanding of SARS-CoV-2 vaccination and mental health and assist governments and policymakers in developing an effective vaccine campaign to achieve vaccination coverage and herd immunity among teachers and staff during the COVID-19 pandemic.

## Materials and methods

### Study design

The study was approved by the Department of Psychology, Jagannath University, Dhaka, Bangladesh, and the Ethics Committee of the First Affiliated Hospital, Zhejiang University School of Medicine, Hangzhou, China before it began. Before the participants started the questionnaire, they had to give their informed consent online. Between March 4 to September 9, 2021, a cross-sectional online study was administered. The data was obtained online using Google Forms and the Bangla language. The five research assistants sent the survey link by e-mail, Facebook, Viber, WhatsApp, Imo, and other social media platforms. They were invited to fill out the form and share the link with their networks to reach more people. They used the snowball method to circulate the survey link throughout their professional and social networks. Participants were told that taking part in the study was entirely voluntary, and they were urged to share the survey link with their colleagues or acquaintances once it was completed. All participants were assured of their data’s privacy and confidentiality, as well as information on the study’s goal, protocol, and their right to have their data removed at any time. The current study received a total of 1,551 responses at the onset. After screening, 24 responses were eliminated due to missing information and being outside of Bangladesh. Overall, data were collected from more than 30 colleges and universities. Finally, responses from 1,527 teachers were included in this study. Six hundred seventy-eight teachers had been vaccinated, and 849 had not. Vaccinated means they had at least one dose vaccinated. The following were the criteria for inclusion: (1) be at least 18 years old, (2) living in Bangladesh at the time of the COVID-19, (3) willingness to engage in this study *via* online informed consent, (4) completion of the whole questionnaire, and (5) no history of mental health problems.

### Participants

The sample size was calculated using OpenEpi software. We assumed a proportion of 50% of the teachers to have poor mental health. This 50% proportion would provide maximum variance and sample size. At 95% confidence level, 80% power, and 1.5 design effect, we arrived at the sample size of 576. The current study inflated our sample by 10% to account for non-response data, so the final sample size required was 634 participants for each group.

### Measurements

#### Demographic characteristics

The participant’s sex (male or female), age (24–35, 36–45, 46–55, or ≥ 56 years), residence (urban or rural), educational level (Masters or lower degree, MPhil degree, Doctoral degree, or other), the status of marriage, and whether or not they had children were self-reported demographic characteristics. Participants were also asked to conduct online classes (yes or no), work experiences (≤ 1, 1–5, 6–10, 11–15, or ≥ 16 years), physical exercise (yes or no), chronic diseases (yes or no), and smoking habit (yes or no). In addition, this study also investigated whether participants had been infected with COVID-19, whether anyone in their family members, friends, or colleagues had been infected with COVID-19, and whether anyone in their family members, friends, or colleagues had died from COVID-19.

Based on our research questions the frequency of symptoms of psychological distress, depression, anxiety, stress, PTSD, insomnia, and fear was assessed using the Bangla versions of the General Health Questionnaire (GHQ-12), Patient health questionnaire (PHQ-2), Generalized Anxiety Disorder Scale (GAD-2), Perceived Stress Scale (PSS-4), Primary Care PTSD Screen for DSM-5 (PC-PTSD-5), Insomnia Severity Index (ISI), and Fear of COVID-19 Scale (FCV-19S) scales, respectively.

#### General health questionnaire

The Bangla version of the 12-item General Health Questionnaire (GHQ-12) (36, 37) evaluates psychological distress on a four-point Likert scale, with “1” defining never and “4” defining frequently. For a full score of 0–12, each item can be assigned a value of 0 (if option 1 or 2) or 1 (if options 3 and 4). The overall score of ≥ 3 indicated that the person’s mental health status was terrible. The internal consistency was α = 0.80.

#### Patient health questionnaire

The Bangla version of the two-item Patient Health Questionnaire (PHQ-2) (38, 39) evaluates depression symptoms rated on a four-point Likert scale, with “1” defining never and “4” defining almost every day. The total score ranges from 0 to 6. The overall value of ≥ 3 is suggested to indicate a likely diagnosis of significant depression. The internal consistency was α = 0.72.

#### Generalized anxiety disorder scale

The Bangla version of the two-item Generalized Anxiety Disorder scale (GAD-2) (40, 41) evaluates anxiety symptoms on a four-point Likert scale, with “1” defining never and “4” defining almost every day. The total score ranges from 0 to 6. The overall score of ≥ 3 is proposed as revealing a probable anxiety disorder diagnosis. The internal consistency was α = 0.84.

#### Perceived stress scale

The Bangla version of the four-item Perceived Stress Scale (PSS-4) (42, 43) evaluates stress symptoms on a four-point Likert scale, with “1” defining never and “4” defining always. The total score ranges from 0 to 16. A quartile split was used because no official cut-off for the PSS-4 scale was available. The internal consistency was α = 0.71.

#### Primary care post-traumatic stress disorder screen for DSM-5

The Bangla version of the five-item Primary Care PTSD Screen for DSM-5 (PC-PTSD-5) (44, 45) evaluates post-traumatic stress disorder symptoms over the past month by asking five binary questions about re-experiencing, avoidance, physiological reactions, emotional numbness, and trauma-distorted guilt and blame thoughts. This scale was previously used in a Bangladeshi study. The total score ranges from 1 to 5, with a 3 as the cut-off value. The internal consistency was α = 0.75.

#### Insomnia severity index

The Bangla version of the seven-item Insomnia Severity Index (ISI) (46, 47) evaluates the severity of insomnia on a five-point Likert scale, with “0” defining no problem and “4” defining a major problem. The total score ranges from 0 to 28. An overall score of ≥ 8 indicates possible insomnia symptoms in this investigation. The internal consistency was α = 0.74.

#### Fear of COVID-19 scale

The Bangla version of the seven-item Fear of COVID-19 Scale (FCV-19S) (48, 49) evaluates the level of fear associated with COVID-19 on a five-point Likert scale, with “1” defining strongly disagree and “5” defining strongly agree. The total score ranges from 7 to 35. The higher the score indicates, the greater the fear of coronavirus-19. The internal consistency was α = 0.89.

#### Oslo social support scale

The Bangla version of the three-item Oslo Social Support Scale (OSSS-3) was also used to evaluate respondents’ social support (45, 50). The raw scores were added together to create a sum index, ranging from 3 to 14. Social support was labeled as poor, moderate, or strong based on a score of 3–8, 9–11, or 12–14. In this study, the internal consistency was α = 0.79.

#### Statistical analysis

The statistical analyses were run by SPSS version 20.0, and figures were prepared in GraphPad Prism version 9. Categorical data was represented using numbers and percentages. To compare categorical variable variations between groups, Chi-square tests were used. The Kolmogorov–Smirnov test, the Shapiro–Wilk test, and normal Q-Q plots were used to determine the data’s normality. The median of the interquartile range (IQR) of data from non-normal distributions was shown. When comparing non-normally distributed data between two groups, the Mann–Whitney *U*-test was used, and when comparing data between more than two groups, the Kruskal–Wallis-test was used. Spearman correlations were used to compare the mental health outcomes of vaccinated and unvaccinated teachers. In addition, binary logistic regression analysis was used to look into potential predictors of mental health outcomes in both groups. The model fitness test was checked using the Hosmer and Lemeshow goodness of fit test. All of the variables were added in the univariate analysis and then the multivariate analysis only included the significant variables in the univariate analysis. For a single predictor, univariate analysis expressed as crude odds ratio (COR) was used, while multivariate analysis expressed as adjusted odds ratio (AOR) was used for multiple predictors, and all mental health outcomes were considered dependent variables. All analyses were conducted at a 95% confidence level, with *p*-values less than 0.05 considered significant.

## Results

### Demographic characteristics

Finally, 1,527 teachers were enrolled in our study, with 678 (44.4%) being vaccinated and 849 (55.6%) being unvaccinated. The characteristics of the study respondents are shown in Table 1. Vaccinated teachers were significantly more likely to be male (76.0 vs. 24.0%), live in urban areas (81.9 vs. 73.4%), have a doctorate (43.1 vs. 22.5%), be married (89.1 vs. 79.7%), having children (73.5 vs. 51.4%), conducted an online class (94.2 vs. 86.5%), engage in daily physical exercise (33.2 vs. 22.5%), have comorbidities (21.2 vs. 10.1%), smoke (13.4 vs. 7.4%), be infected with COVID-19 (15.5 vs. 10.5%), have family members, friends, or colleagues infected with COVID-19 (86.0 vs. 75.9%) and died from COVID-19 (43.1 vs. 29.9%) than unvaccinated teachers. On the other hand, unvaccinated teachers were substantially more likely to be in the age groups of 36–45 years old (68.1 vs. 32.4%) and also to have 1–5 years of work experience (42.5 vs. 22.0%) than vaccinated teachers. Furthermore, there were no significant differences in social support between vaccinated and unvaccinated teachers (*p* = 0.51).

**TABLE 1:** Demographic characteristics in vaccinated and unvaccinated teachers against COVID-19 infection.

Characteristics	Total (*n* = 1,527)	Vaccinated teachers (*n* = 678)	Unvaccinated teachers (*n* = 849)	*P*-value
	
	No. (%)	No. (%)	No. (%)	
**Sex**				
Male	1,081 (70.8)	515 (76.0)	566 (66.7)	0.00
Female	446 (29.2)	163 (24.0)	283 (33.3)	
**Age, y**				
24–35	798 (52.3)	220 (32.4)	578 (68.1)	0.00
36–45	451 (29.5)	237 (35.0)	214 (25.2)	
46–55	204 (13.4)	158 (23.3)	46 (5.4)	
≥ 56	74 (4.8)	63 (9.3)	11 (1.3)	
**Residence**				
Urban	1,178 (77.1)	555 (81.9)	623 (73.4)	0.00
Rural	349 (22.9)	123 (18.1)	226 (26.6)	
**Education level**				
Masters or lower degree	853 (55.9)	266 (39.2)	587 (69.1)	0.00
MPhil degree	67 (4.4)	27 (4.0)	40 (4.7)	
Doctoral degree	483 (31.6)	292 (43.1)	191 (22.5)	
Other	124 (8.1)	93 (13.7)	31 (3.7)	
**Marital status**				
Single	231 (15.1)	64 (9.4)	167 (19.7)	0.00
Married	1,281 (83.9)	604 (89.1)	677 (79.7)	
Divorced/separated/widowed	15 (1.0)	10 (1.5)	5 (0.6)	
**Having children**				
Yes	934 (61.2)	498 (73.5)	436 (51.4)	0.00
No	593 (38.8)	180 (26.5)	413 (48.6)	
**Do you have conducted an online class?**				
Yes	1,373 (89.9)	639 (94.2)	734 (86.5)	0.00
No	154 (10.1)	39 (5.8)	115 (13.5)	
**Work experiences, y**				
< 1	52 (3.4)	6 (0.9)	46 (5.4)	0.00
1–5	510 (33.4)	149 (22.0)	361 (42.5)	
6–10	331 (21.7)	115 (17.0)	216 (25.4)	
11–15	268 (17.6)	122 (18.0)	146 (17.2)	
≥ 16	366 (24.0)	286 (42.2)	80 (9.4)	
**Physical exercise**				
Yes	416 (27.2)	225 (33.2)	191 (22.5)	0.00
No	1,111 (72.8)	453 (66.8)	658 (77.5)	
**Chronic diseases**				
Yes	230 (15.1)	144 (21.2)	86 (10.1)	0.00
No	1,297 (84.9)	534 (78.8)	763 (89.9)	
**Smoking habit**				
Yes	154 (10.1)	91 (13.4)	63 (7.4)	0.00
No	1,373 (89.9)	587 (86.6)	786 (92.6)	
**Personal COVID-19 infection**				
Yes	194 (12.7)	105 (15.5)	89 (10.5)	0.00
No	1,333 (87.3)	573 (84.5)	760 (89.5)	
**Have any of your family members, friends, or colleagues been infected with the COVID-19?**				
Yes	1,227 (80.4)	583 (86.0)	644 (75.9)	0.00
No	300 (19.6)	95 (14.0)	205 (24.1)	
**Have any of your family members, friends, or colleagues died of the COVID-19?**				
Yes	546 (35.8)	292 (43.1)	254 (29.9)	0.00
No	981 (64.2)	386 (56.9)	595 (70.1)	
**Social support**				
Poor	438 (28.7)	185 (27.3)	253 (29.8)	0.51
Moderate	620 (40.6)	284 (41.9)	336 (39.6)	
Strong	469 (30.7)	209 (30.8)	260 (30.6)	

### Prevalence of mental health outcomes

**FIGURE 1:**
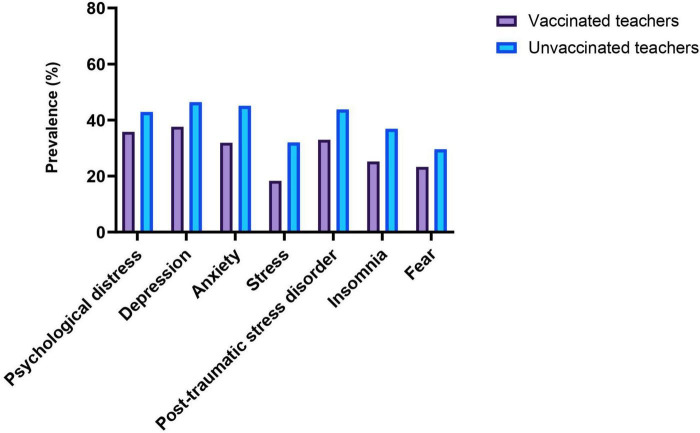
Prevalence of mental health outcomes among vaccinated and unvaccinated teachers against COVID-19 infection.

The prevalence of mental health outcomes among vaccinated and unvaccinated teachers against COVID-19 infection are shown in Figure 1 and Table 2. The prevalence rates of symptoms of psychological distress, depression, anxiety, stress, PTSD, insomnia, and fear symptoms among vaccinated teachers were 35.8, 37.6, 31.9, 18.3, 33.0, 25.2, and 23.3%, respectively. On the other hand, the prevalence rates of symptoms of psychological distress, depression, anxiety, stress, PTSD, insomnia, and fear symptoms among unvaccinated teachers were 42.9, 46.4, 45.1, 32.0, 43.8, 36.9, and 29.6%, respectively. However, vaccinated teachers had a significantly lower prevalence rates of psychological distress (35.8 vs. 42.9%), depression (37.6 vs. 46.4%), anxiety (31.9 vs. 45.1%), stress (18.3 vs. 32.0%), PTSD (33.0 vs. 43.8%), insomnia (25.2 vs. 36.9%), and fear symptoms (23.3 vs. 29.6%) compared to unvaccinated teachers.

**TABLE 2:** The prevalence of mental health outcomes among vaccinated and unvaccinated teachers against COVID-19 infection.

Mental health outcomes	Total (*n* = 1,527)	Vaccinated teachers (*n* = 678)	Unvaccinated teachers (*n* = 849)	*P*-value
	
	No. (%)	No. (%)	No. (%)	
**Psychological distress symptoms**				
Yes	607 (39.8)	243 (35.8)	364 (42.9)	0.00
No	920 (60.2)	435 (64.2)	485 (57.1)	
**Depression symptoms**				
Yes	649 (42.5)	255 (37.6)	394 (46.4)	0.00
No	878 (57.5)	423 (62.4)	455 (53.6)	
**Anxiety symptoms**				
Yes	599 (39.2)	216 (31.9)	383 (45.1)	0.00
No	928 (60.8)	462 (68.1)	466 (54.9)	
**Stress symptoms**				
Yes	396 (25.9)	124 (18.3)	272 (32.0)	0.00
No	1,131 (74.1)	554 (81.7)	577 (68.0)	
**PTSD symptoms**				
Yes	596 (39.0)	224 (33.0)	372 (43.8)	0.00
No	931 (61.0)	454 (67.0)	477 (56.2)	
**Insomnia symptoms**				
Yes	484 (31.7)	171 (25.2)	313 (36.9)	0.00
No	1,043 (68.3)	507 (74.8)	536 (63.1)	
**Fear symptoms**				
Yes	409 (26.8)	158 (23.3)	251 (29.6)	0.00
No	1,118 (73.2)	520 (76.7)	598 (70.4)	

PTSD, Post-traumatic stress disorder.

### Scores of mental health outcomes

**FIGURE 2:**
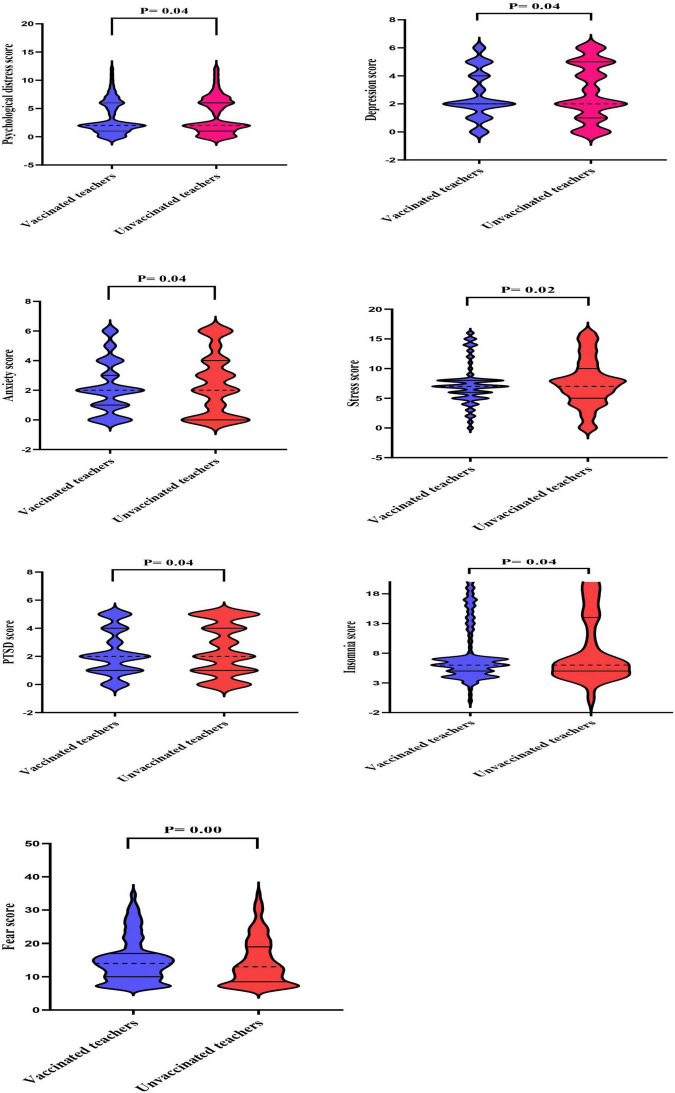
The median of the interquartile range (IQR) of mental health outcome scores in vaccinated and unvaccinated teachers against COVID-19 infection. Violin plots reveal medians (dotted lines) and interquartile ranges (solid lines).

As shown in Figure 2, violin plots revealed that unvaccinated teachers had significantly lower median of the IQR of scores for fear symptoms compared to vaccinated teachers (13.0 [8.5–19.0] vs. 14.0 [10.0–17.0]). The median scores for the remaining symptoms were significantly the same.

### Correlations of mental health outcomes

Spearman’s correlations of psychological outcomes among vaccinated and unvaccinated teachers are shown in Table 3. In the vaccinated teachers, there was a significantly negative correlation between psychological distress scores and insomnia (r_*s*_ = −0.123) scores. Moreover, depression scores were significantly positively linked to anxiety (r_*s*_ = 0.333), stress (r_*s*_ = 0.183), insomnia (r_*s*_ = 0.225) and fear (r_*s*_ = 0.373) scores. Furthermore, there was a significantly positive relationship between anxiety scores and stress (r_*s*_ = 0.257), PTSD (r_*s*_ = 0.160), insomnia (r_*s*_ = 0.263) and fear (r_*s*_ = 0.424) scores. However, stress scores were significantly positively linked to PTSD (r_*s*_ = 0.132), insomnia (r_*s*_ = 0.154) and fear (r_*s*_ = 0.325) scores. In addition, we also discovered a significantly positive link between PTSD and fear (r_*s*_ = 0.128) scores, as well as a link between insomnia and fear (r_*s*_ = 0.305) scores.

**TABLE 3:** Spearman’s correlations of mental health outcomes among vaccinated and unvaccinated teachers against COVID-19 infection.

Teachers	Mental health outcomes	1	2	3	4	5	6	7
**Vaccinated teachers**	**1**	1.00						
	**2**	0.025	1.00					
	**3**	–0.009	0.333**	1.00				
	**4**	–0.058	0.183**	0.257**	1.00			
	**5**	0.038	0.064	0.160**	0.132**	1.00		
	**6**	−0.123**	0.225**	0.263**	0.154**	0.067	1.00	
	**7**	–0.034	0.373**	0.424**	0.325**	0.128**	0.305**	1.00
**Unvaccinated teachers**	**1**	1.00						
	**2**	–0.037	1.00					
	**3**	–0.045	0.533**	1.00				
	**4**	−0.112**	0.310**	0.470**	1.00			
	**5**	−0.023**	–0.047	–0.045	–0.040	1.00		
	**6**	−0.069*	0.293**	0.404**	0.300**	0.032	1.00	
	**7**	–0.038	0.374**	0.568**	0.469**	–0.059	0.381**	1.00

*p < 0.05, **p < 0.01. 1 Psychological distress, 2 Depression, 3 Anxiety, 4 Stress, 5 Post-traumatic stress disorder, 6 Insomnia, 7 Fear.

In the unvaccinated teachers, psychological distress scores were significantly negatively linked to stress (r_*s*_ = −0.112), PTSD (r_*s*_ = −0.023), and insomnia (r_*s*_ = −0.069) scores. Moreover, depression scores were significantly positively linked to anxiety (r_*s*_ = 0.533), stress (r_*s*_ = 0.310), insomnia (r_*s*_ = 0.293) and fear (r_*s*_ = 0.374) scores. Furthermore, there was a significantly positive relationship between anxiety scores and stress (r_*s*_ = 0.470), insomnia (r_*s*_ = 0.404) and fear (r_*s*_ = 0.568) scores. However, stress scores were significantly positively linked to insomnia (r_*s*_ = 0.300) and fear (r_*s*_ = 0.469) scores. In addition, we also discovered a significantly positive link between insomnia and fear (r_*s*_ = 0.381) scores.

### Risk factors of mental health outcomes

The results of the univariate logistic regression analysis of mental health outcomes and associated factors among vaccinated and unvaccinated teachers against COVID-19 infection are presented in Supplementary Table 1. The variables found to be significant in the univariate logistic regression analysis were included in the multivariate analysis. The multivariate logistic regression analysis (Supplementary Table 2) showed that vaccinated teachers with master’s or lower degree levels had significantly higher symptoms of depression (AOR, 1.31; 95% CI, 1.02–1.62), stress (AOR, 1.44; 95% CI, 1.21–3.12), and fear (AOR, 1.50; 95% CI, 1.14–3.26) than other education levels. Respondents with children had a significantly higher risk of depression (AOR, 1.39; 95% CI, 1.08–2.33), anxiety (AOR, 1.24; 95% CI, 1.02–2.13), stress (AOR, 1.41; 95% CI, 1.18–2.26), and fear (AOR, 1.49; 95% CI, 1.12–1.87) symptoms than those who did not have children. Compared to those who had worked for more than sixteen years, those who had worked for less than 5 years were significantly more likely to experience symptoms of depression (AOR, 1.80; 95% CI, 1.34–2.86), anxiety (AOR, 1.68; 95% CI, 1.17–5.89), and stress (AOR, 1.89; 95% CI, 1.28–2.76). Respondents who lost family members, friends, or colleagues due to the COVID-19 pandemic had a significantly higher chance of experiencing symptoms of anxiety (AOR, 1.58; 95% CI, 1.27–2.84), PTSD (AOR, 1.29; 95% CI, 1.02–1.48), and fear (AOR, 1.44; 95% CI, 1.05–1.88) than those who did not. Compared to participants with strong social support, those with poor social support had a higher risk of psychological distress (AOR, 2.42; 95% CI, 1.55–3.80) and depression (AOR, 1.59; 95% CI, 1.37–2.92) symptoms, but a lower risk of anxiety (AOR, 0.63; 95% CI, 0.26–0.99), stress (AOR, 0.77; 95% CI, 0.24–0.90), and insomnia (AOR, 0.71; 95% CI, 0.20–0.91) symptoms.

On the other hand, unvaccinated male teachers were significantly associated with a higher risk of all mental health outcomes except psychological distress and PTSD symptoms compared to female teachers (e.g., depression: AOR, 1.55; 95% CI, 1.11–2.15; anxiety: AOR, 1.72; 95% CI, 1.23–2.40; fear: AOR, 1.78; 95% CI, 1.24–2.56). Participants who exercised daily had substantially less likely to suffer from psychological distress (AOR, 0.70; 95% CI, 0.49–1.00), anxiety (AOR, 0.80; 95% CI, 0.22–0.91), and stress (AOR, 0.37; 95% CI, 0.11–0.88) symptoms than those who did not. Compared to those who did not have chronic diseases, those with chronic diseases were significantly less likely to experience symptoms of depression (AOR, 0.51; 95% CI, 0.32–0.83), anxiety (AOR, 0.44; 95% CI, 0.27–0.73), stress (AOR, 0.47; 95% CI, 0.28–0.79), and fear (AOR, 0.32; 95% CI, 0.19–0.53). Participants who were smokers had a significantly higher chance of anxiety (AOR, 1.35; 95% CI, 1.19–2.63), stress (AOR, 1.41; 95% CI, 1.08–1.74), and fear (AOR, 1.38; 95% CI, 1.07–2.70) symptoms than non-smokers. Compared to participants with strong social support, those with poor social support had a higher risk of all mental health outcomes except PTSD symptoms (e.g., psychological distress: AOR, 2.32; 95% CI, 1.58–3.43; anxiety: AOR, 1.36; 95% CI, 1.03–1.64; fear: AOR, 1.86; 95% CI, 1.08–2.58).

## Discussion

This is the first study in Bangladesh to compare the mental health outcomes and variables associated with COVID-19 infection among vaccinated and unvaccinated teachers. The questionnaire was completed by 1,527 Bangladeshi college and university teachers, with 678 (44.4%) opting to be vaccinated. At least one dose of COVID-19 vaccine from Oxford-AstraZeneca, Pfizer–BioNTech, Sinopharm, or Moderna was administered to the vaccinated teachers. In our survey, 849 (55.6%) teachers had not been immunized. Generally, teachers are well educated and conscious about their health and safety. But the phenomenon is different in Bangladesh. First, there could be a reluctance or refusal to take the vaccine as soon as possible. Few studies have been conducted in Bangladesh to assess the COVID-19 vaccine hesitancy, which has reported a vaccine hesitancy rate between 25.4 and 50%. It might be possible that lack of knowledge related to the vaccine, conspiracy beliefs regarding the origin, effectiveness, and consequences of receiving vaccines, newness, safety, and probable side effects, etc (51–53). At the beginning of June 2021, less than four percent of the Bangladesh population had received two doses (30). Second, during our data collection time, all the educational institutions closed as a precautionary measure against coronavirus. So that unavailability of another reason here. Third, another reason might be that 52.3% of the teachers were young, aged 24–35, and the rest of the 47.7% were older in the present survey. The vaccine was given to older people, like 55 years up, and then gradually, age issues were waived. So during this period, younger teachers could not be more vaccinated. From this information, it is clear that taking vaccines was not easier during the data collection period in Bangladesh.

However, our study revealed that vaccinated teachers had a lower prevalence of psychological outcomes than unvaccinated teachers against the COVID-19 outbreak in Bangladesh. These findings paralleled a study conducted in the United States among 453,167 adults, which found that those who had been vaccinated had 17% lower odds of depression and 13% lower odds of anxiety than those who had not been vaccinated against the COVID-19 outbreak (54). A study conducted in China among 4,244 individuals reported that the COVID-19 vaccine could improve the mental health status of vaccinated individuals (55), while another study conducted between January 29 to April 26, 2021, in the same country found that being vaccinated against the COVID-19 outbreak was linked to a lower risk of psychological stress (34). Moreover, Koltai et al. (56) study was done between March 2020 to June 2021 and reported that being vaccinated for COVID-19 was associated with declines in psychological distress than those not vaccinated. Furthermore, a study conducted in Turkey among 304 individuals by Bilge et al. (57) found that the vaccinated individuals had lower scores for depression and anxiety symptoms than unvaccinated individuals, indicating that vaccination may have a positive effect on improving mental health. Our hypotheses were partially confirmed or positively correlated based on the information presented above. The current study discovered many factors linked to both vaccinated and unvaccinated teachers.

Our findings showed that vaccinated teachers with master’s or lower degree levels were significantly more likely to experience depression, stress, and fear symptoms. This finding is consistent with previous research, which found that teachers with a master’s or lower degree level were poorer psychological outcomes before the COVID-19 pandemic (6) and were more accepting of COVID-19 vaccination (58). Studies conducted in 19 countries around the world and the United States found that the number of years of education was linked to increased acceptance of the COVID-19 vaccine (59). In Australia, a lack of willingness to be vaccinated against SARS-CoV-2 is linked to lower levels of education (60). It could be attributed to widespread vaccine propaganda spread through various channels (61).

Our findings revealed that vaccinated teachers with children had a significantly higher risk of depression, anxiety, stress, and fear symptoms. Similar findings are found in a recent study of 1,633 teachers in northern Spain, which found that teachers who had children during the COVID-19 pandemic had more depressive symptoms than those who did not (62). Similarly, in a study of 394 teachers in Ecuador, Hidalgo-Andrade et al. (63) revealed that teachers with children had a higher risk of perceived stress symptoms during the COVID-19 pandemic. Moreover, it is also consistent with earlier studies conducted among 2,665 teachers, which found that teachers with children were more likely to experience fear symptoms during the COVID-19 pandemic (64). Not only during the COVID-19 pandemic but also before the outbreak, teachers with children were more likely to report depression, anxiety, and stress symptoms (6). However, this result also corresponds to other studies, which found that teachers who are also parents have positive attitudes toward receiving the SARS-CoV-2 vaccine (65). It’s possible that participants were worried about the COVID-19 pandemic effects on themselves or their children. Therefore, they may desire to protect themselves or their children by getting COVID-19 vaccines.

The present study found that working for less than 5 years was a higher risk factor for symptoms of depression, anxiety, and stress among vaccinated teachers, which is in agreement with prior studies that found teachers with less than 5 years of work experience were associated poorer mental health during the coronavirus outbreak (66). Another study conducted before the pandemic discovered that teachers with more than 3 years of experience in the classroom had higher stress levels (67). However, our findings contradict a recent study involving 399 Greek teachers, which found that teachers with more than fifteen years of work experience were higher likely to receive the SARS-CoV-2 vaccine (68). It could be the reason for variations in the study population, research procedure, vaccine timeline, and socio-cultural factors of the study participants.

The present research results have also shown that vaccinated teachers who lost family members, friends, or colleagues due to the COVID-19 pandemic had a significantly higher chance of experiencing anxiety, PTSD, and fear symptoms. This finding is consistent with recent research by Orrù et al. (69), who found that respondents who had lost one or more relatives due to the COVID-19 pandemic had higher levels of anxiety and fear symptoms. Similarly, an online survey involving 1,650 college teachers was conducted from April 26 to April 29, 2020, and discovered that those who had family members or relatives die due to the COVID-19 pandemic had an increased chance of PTSD symptoms than those who had anyone die (11). However, our findings contradict previous research, which found that participants’ willingness or hesitancy to take the coronavirus vaccine was not significantly related to having a close friend or relative die of coronavirus (70). This may be due to an increased potential risk of coronavirus infection among our study participants. Previous studies have found that people who perceive a high risk of contracting coronavirus were higher likely to pay for pandemic vaccination (71). People are rational, so if they believe they are in danger, they will take steps to mitigate the risk. It could explain why people who believed coronavirus was a high-risk disease were more willing to spend money on the vaccine.

Our findings revealed that poor social support was a higher risk factor for psychological distress and depression symptoms, but a lower risk factor for anxiety, stress, and insomnia symptoms among vaccinated teachers. This finding is supported by the results of prior studies (72, 73). In a study of 231 educators and health professionals, Khan et al. (74) discovered that social support is negatively associated with depression, anxiety, and stress symptoms among educators. A recent study carried out among 2,020 individuals in Lebanon during the COVID-19 pandemic revealed that low levels of social support were associated with a higher risk of depression, and poor sleep quality symptoms (75). However, both of the above results are in some ways in conflict with our findings. However, our results are inconsistent with other studies conducted among 435 adult women in the Philippines, which identified that social support was linked to a positive intention to receive the human papillomavirus (HPV) vaccine (76). A variety of factors could contribute to differences in study participants, research procedures, measurement methods, vaccine types, and cultures.

Our findings showed that unvaccinated male teachers were significantly associated with an increased risk of all mental health outcomes except psychological distress and PTSD symptoms than female teachers. This finding is inconsistent with other pandemic studies (63). A study in Spain among 1,633 teachers reported that women exhibit significantly more symptoms of stress and anxiety than men (62). Similar scenarios were discovered in pre-pandemic studies (6). Moreover, a rapid systematic review with meta-analysis found no significant differences in stress or anxiety between male and female teachers. Studies measuring depression did not differ on this variable (10). Our findings are also not supported by prior studies that found male teachers were higher likely to pay for the SARS-CoV-2 vaccine than female teachers (71, 77). Our results are also consistent with those of an earlier study in Indonesia on attitudes toward dengue vaccination (78) and a study in Bangladesh on attitudes toward the COVID-19 vaccine (79). Females may be more likely than males to favorable opinion toward the COVID-19 vaccine. Therefore, males are less likely to accept the COVID-19 vaccine (80).

Our findings discovered that unvaccinated teachers who exercised daily had a significantly lower risk of psychological distress, anxiety, and stress symptoms, which is in agreement with prior Bangladeshi studies that found participants who did not or insufficiently engage in physical activity were more likely to experience symptoms of stress and anxiety during the coronavirus pandemic (81). Similarly, a study of 663 teachers in Turkey found that those who exercised regularly during the COVID-19 pandemic had less anxiety and were in better health than those who did not (82). Furthermore, pre-pandemic research yielded similar results (67). However, a large-scale cross-sectional survey in China found that people who never exercised regularly accepted the COVID-19 vaccine more freely, which is consistent with our findings (77). They may believe their immune systems are robust because they exercise regularly, so they do not require the COVID-19 vaccine. Whether they exercise daily or not, the current study recommends getting vaccinated as soon as possible.

The present study found that unvaccinated teachers with chronic diseases had a significantly lower risk of symptoms of depression, anxiety, stress, and fear. A study conducted among 1,633 Spanish teachers between September 5 to September 28, 2020, found that the teachers with chronic diseases or those who live with others with chronic diseases have more depression, anxiety, and stress symptoms during the coronavirus pandemic, which is inconsistent with our results (62). Our findings also contradicted the findings of Al-Rahimi et al. (83), who claimed that during the COVID-19 outbreak, a significant number of people with chronic diseases felt anxiety and fear symptoms. According to a Bangladeshi study, participants with chronic diseases were found to have higher vaccine hesitancy rates, which is supported by our findings (84). However, they need to be conscious about their health and try to be vaccinated as soon as possible.

The present study suggests that unvaccinated teachers with smokers had a significantly higher risk of anxiety, stress, and fear symptoms, which is consistent with recent Bangladeshi studies (85). Moreover, according to a study conducted in Bangladesh, those who smoked more frequently had significantly higher stress symptoms during the coronavirus pandemic (86). Furthermore, a pre-pandemic study of 41 low-middle-income countries found that people with higher stress levels were more likely to smoke (87), which is also supported by our results. Nguyen et al. (88) revealed that respondents with a higher fear score were more likely to smoke during the COVID-19 outbreak, which is also in line with our findings. However, this result also corresponds to other studies, which found that people who had previously smoked were more highly probable to be skeptical of the COVID-19 vaccine (89, 90). Similarly, a national cross-sectional study in China found that people who had smoked were more likely to be vaccinated (91). Smoking and vaccination have also been linked in the case of other viruses (e.g., influenza) (92). Some smokers think that smoking has little or no impact on the risk of developing severe coronavirus infections (93). We are concerned that if this potential protective effect is confirmed, smokers will interpret it as a sign that the vaccine is ineffective (90). In addition, smokers are more likely to involve in other health-damaging behaviors, resulting in low vaccine acceptance (92). Governments and policymakers should educate the public about the benefits of smoking cessation vaccination and debunk the myth that smoking protects against COVID-19 (94).

It was not surprising that poor social support had a significantly higher risk factor for all the mental health outcomes except PTSD symptoms among unvaccinated teachers. The majority of pre-pandemic studies have found that social support is linked to better mental health outcomes (95, 96). Moreover, during the coronavirus pandemic, a study of 751 teachers in China discovered that social support can relieve acute stress symptoms when it meets individuals’ psychological needs and improves their sense of control (97). Another study involving 681 French participants discovered that poor social support was linked to increased depression symptoms (98). Furthermore, our results were consistent with those of Yenen and Çarkit (12), who discovered that in 322 Turkish teachers, lower perceived social support was linked to a higher fear of COVID-19. However, a study in the United Kingdom reported that social support appears to be linked to an increased likelihood of COVID-19 testing and vaccination, which is also supported by our results. Social support appears to be crucial not only for addressing mental health outcomes but may also be linked to vaccine antibody responses (99, 100). Therefore, the findings of this study may inform teachers with low social support that increasing family, co-worker, and workplace support may improve mental health problems and decrease aversion to the COVID-19 vaccine.

## Strengths and limitations

The following are some of the study’s advantages: first, the first study in Bangladesh that has evaluated the mental health outcomes and associated factors among vaccinated and unvaccinated teachers against COVID-19 infection. Second, this innovative research discovered that teachers vaccinated against COVID-19 infection had a significant positive effect on their mental health. Third, it was possible to draw meaningful conclusions from this study because it included a diverse group of college and university teachers. Fourth, this research will add to our understanding of COVID-19 vaccination and mental health, as well as assist governments and policymakers in developing an effective vaccine campaign to achieve vaccination coverage and herd immunity among teachers and staff during the COVID-19 pandemic. Finally, this study could help teachers, students, parents, and other professionals develop positive attitudes toward vaccination.

This study provides novel findings on mental health outcomes and associated factors among vaccinated and unvaccinated Bangladeshi teachers against COVID-19 infection, but its limitations must not be overlooked. First, mental health outcomes were determined using a self-report tool and an online survey. Future research should include clinical interviews or qualitative studies to get a more complete picture of the problem. Second, because it was a cross-sectional study, there was no way to prove causation. As a result, this study recommends that longitudinal studies be conducted to overcome this limitation. Third, snowball sampling was used in this study, resulting in selection biases and poor representativeness. Fourth, it is impossible to assess the participation rate because it is unknown how many subjects received the survey link. Fifth, the factors related to unvaccinated (e.g., willingness, hesitancy, and shortage of vaccine) were unclear. Finally, this study did not consider influencing factors such as socioeconomic status, family history of mental disorders, and taking any vaccine after the age of 18.

## Conclusion

We observed a lower prevalence of mental health outcomes among vaccinated teachers than in unvaccinated teachers against COVID-19 infection in Bangladesh. This study suggests emphasizing the vaccinated to unvaccinated teachers as soon as possible to control the infection and improve mental health outcomes. Vulnerable teachers also required special attention, health-related education, and psychological support.

## Data availability statement

The original contributions presented in the study are included in the article/Supplementary material, further inquiries can be directed to the corresponding author.

## Ethics statement

The studies involving human participants were reviewed and approved by Department of Psychology, Jagannath University, Dhaka, Bangladesh, and the Ethics Committee of the First Affiliated Hospital, Zhejiang University School of Medicine, Hangzhou, China. The patients/participants provided their written informed consent to participate in this study.

## Author contributions

MA: conceptualization, methodology, formal analysis, and writing—original draft. MA, AI, MSH, AH, DA, and MMH: data collection. MA and YX: writing—review, and editing. All authors read and approved the final manuscript.
