# Supplementary material for: Mental health outcomes and associated factors among vaccinated and unvaccinated teachers against COVID-19 infection in Bangladesh
Source: Front Psychiatry. 2022 Aug 3;13:896419. doi: 10.3389/fpsyt.2022.896419 (PMC9382101; doi:10.3389/fpsyt.2022.896419)
Supplement: Supplementary file 1 [file Table_1.docx]

**Supplement Table S1.** Univariate regression analysis of factors associated with mental health outcomes among vaccinated and unvaccinated teachers against COVID-19 infection.

| **Variables** | **COR (95% CI)** | ***p* value** |
| --- | --- | --- |
| ***Models for psychological distress symptoms*** |  |  |
| **Vaccinated teachers** |  |  |
| Sex |  |  |
| Male | 0.63 (0.43-0.93) | 0.01 |
| Female | 1 [Reference] |  |
| Social support |  |  |
| Poor | 2.59 (1.69-3.96) | 0.00 |
| Moderate | 1.92 (1.33-2.77) | 0.00 |
| Strong | 1 [Reference] |  |
| **Unvaccinated teachers** |  |  |
| Age, y |  |  |
| 24-35 | 3.75 (1.74-14.3) | 0.05 |
| 36-45 | 3.89 (1.00-15.0) | 0.04 |
| 46-55 | 1.56 (1.01-6.70) | 0.54 |
| ≥56 | 1 [Reference] |  |
| Work experiences, y |  |  |
| <1 | 1.31 (0.63-2.72) | 0.46 |
| 1-5 | 1.40 (0.86-2.28) | 0.17 |
| 6-10 | 1.91 (1.14-3.22) | 0.01 |
| 11-15 | 1.45 (0.84-2.51) | 0.17 |
| ≥16 | 1 [Reference] |  |
| Physical exercise |  |  |
| Yes | 0.56 (0.40-0.77) | 0.00 |
| No | 1 [Reference] |  |
| Social support |  |  |
| Poor | 2.58 (1.80-3.71) | 0.00 |
| Moderate | 1.42 (1.02-1.96) | 0.03 |
| Strong | 1 [Reference] |  |
| ***Models for depression symptoms*** |  |  |
| **Vaccinated teachers** |  |  |
| Sex |  |  |
| Male | 1.87 (1.31-2.67) | 0.00 |
| Female | 1 [Reference] |  |
| Education level |  |  |
| Masters or lower degree | 0.56 (0.34-0.93) | 0.02 |
| MPhil degree | 0.62 (0.25-1.52) | 0.30 |
| Doctoral degree | 0.81 (0.49-1.34) | 0.42 |
| Other | 1 [Reference] |  |
| Having children |  |  |
| Yes | 1.57 (1.11-2.22) | 0.01 |
| No | 1 [Reference] |  |
| Work experiences, y |  |  |
| <1 | 0.47 (0.09-2.39) | 0.36 |
| 1-5 | 1.59 (1.04-1.90) | 0.01 |
| 6-10 | 0.68 (0.43-1.07) | 0.09 |
| 11-15 | 0.75 (0.48-1.17) | 0.21 |
| ≥16 | 1 [Reference] |  |
| Have any of your family members, friends, or colleagues been infected with the COVID-19? |  |  |
| Yes | 0.48 (0.29-0.79) | 0.00 |
| No | 1 [Reference] |  |
| Social support |  |  |
| Poor | 1.57 (1.14-2.87) | 0.01 |
| Moderate | 1.42 (1.07-1.81) | 0.00 |
| Strong | 1 [Reference] |  |
| **Unvaccinated teachers** |  |  |
| Sex |  |  |
| Male | 1.55 (1.16-2.07) | 0.00 |
| Female | 1 [Reference] |  |
| Having children |  |  |
| Yes | 1.39 (1.06-1.82) | 0.01 |
| No | 1 [Reference] |  |
| Work experiences, y |  |  |
| <1 | 0.58 (0.28-1.23) | 0.15 |
| 1-5 | 1.60 (1.26-1.90) | 0.05 |
| 6-10 | 0.50 (0.29-0.85) | 0.01 |
| 11-15 | 0.73 (0.41-1.28) | 0.27 |
| ≥16 | 1 [Reference] |  |
| Chronic diseases |  |  |
| Yes | 0.59 (0.37-0.92) | 0.02 |
| No | 1 [Reference] |  |
| Social support |  |  |
| Poor | 0.71 (0.38-0.97) | 0.00 |
| Moderate | 0.56 (0.40-0.78) | 0.00 |
| Strong | 1 [Reference] |  |
| ***Models for anxiety symptoms*** |  |  |
| **Vaccinated teachers** |  |  |
| Sex |  |  |
| Male | 1.65 (1.14-2.38) | 0.00 |
| Female | 1 [Reference] |  |
| Chronic diseases |  |  |
| Yes | 0.60 (0.41-0.87) | 0.00 |
| No | 1 [Reference] |  |
| Have any of your family members, friends, or colleagues been infected with the COVID-19? |  |  |
| Yes | 0.60 (0.36-1.00) | 0.05 |
| No | 1 [Reference] |  |
| Have any of your family members, friends, or colleagues died of the COVID-19? |  |  |
| Yes | 0.64 (0.46-0.89) | 0.00 |
| No | 1 [Reference] |  |
| Social support |  |  |
| Poor | 0.49 (0.31-0.77) | 0.00 |
| Moderate | 0.44 (0.29-0.66) | 0.00 |
| Strong | 1 [Reference] |  |
| **Unvaccinated teachers** |  |  |
| Sex |  |  |
| Male | 1.79 (1.34-2.39) | 0.00 |
| Female | 1 [Reference] |  |
| Education level |  |  |
| Masters or lower degree | 0.43 (0.19-0.96) | 0.04 |
| MPhil degree | 0.50 (0.18-1.35) | 0.17 |
| Doctoral degree | 0.67 (0.29-1.54) | 0.35 |
| Other | 1 [Reference] |  |
| Having children |  |  |
| Yes | 1.32 (1.00-1.73) | 0.04 |
| No | 1 [Reference] |  |
| Work experiences, y |  |  |
| <1 | 0.32 (0.14-0.73) | 0.00 |
| 1-5 | 0.24 (0.13-0.44) | 0.00 |
| 6-10 | 0.21 (0.11-0.40) | 0.00 |
| 11-15 | 0.31 (0.16-0.59) | 0.00 |
| ≥16 | 1 [Reference] |  |
| Physical exercise |  |  |
| Yes | 2.45 (1.73-3.48) | 0.00 |
| No | 1 [Reference] |  |
| Chronic diseases |  |  |
| Yes | 0.52 (0.33-0.83) | 0.00 |
| No | 1 [Reference] |  |
| Smoking habit |  |  |
| Yes | 0.41 (0.24-0.70) | 0.00 |
| No | 1 [Reference] |  |
| Social support |  |  |
| Poor | 0.35 (0.25-0.51) | 0.00 |
| Moderate | 0.49 (0.35-0.69) | 0.00 |
| Strong | 1 [Reference] |  |
| ***Models for stress symptoms*** |  |  |
| **Vaccinated teachers** |  |  |
| Sex |  |  |
| Male | 1.59 (1.03-2.44) | 0.03 |
| Female | 1 [Reference] |  |
| Age, y |  |  |
| 24-35 | 0.46 (0.21-1.00) | 0.05 |
| 36-45 | 0.96 (0.43-2.12) | 0.92 |
| 46-55 | 1.03 (0.44-2.38) | 0.94 |
| ≥56 | 1 [Reference] |  |
| Education level |  |  |
| Masters or lower degree | 1.35 (1.03-3.70) | 0.00 |
| MPhil degree | 1.07 (0.27-4.16) | 0.91 |
| Doctoral degree | 0.92 (0.45-1.89) | 0.83 |
| Other | 1 [Reference] |  |
| Having children |  |  |
| Yes | 2.01 (1.33-3.04) | 0.00 |
| No | 1 [Reference] |  |
| Do you have conducted an online class? |  |  |
| Yes | 2.69 (1.35-5.34) | 0.00 |
| No | 1 [Reference] |  |
| Work experiences, y |  |  |
| <1 | 0.15 (0.03-0.81) | 0.02 |
| 1-5 | 0.44 (0.27-0.73) | 0.00 |
| 6-10 | 0.56 (0.32-0.99) | 0.04 |
| 11-15 | 0.91 (0.49-1.66) | 0.76 |
| ≥16 | 1 [Reference] |  |
| Social support |  |  |
| Poor | 0.36 (0.21-0.62) | 0.00 |
| Moderate | 0.65 (0.38-1.09) | 0.10 |
| Strong | 1 [Reference] |  |
| **Unvaccinated teachers** |  |  |
| Sex |  |  |
| Male | 1.51 (1.12-2.04) | 0.00 |
| Female | 1 [Reference] |  |
| Education level |  |  |
| Masters or lower degree | 0.28 (0.10-0.83) | 0.02 |
| MPhil degree | 0.22 (0.06-0.75) | 0.01 |
| Doctoral degree | 0.38 (0.12-1.15) | 0.08 |
| Other | 1 [Reference] |  |
| Having children |  |  |
| Yes | 1.91 (1.42-2.56) | 0.00 |
| No | 1 [Reference] |  |
| Work experiences, y |  |  |
| <1 | 0.40 (0.15-0.89) | 0.05 |
| 1-5 | 0.23 (0.11-0.47) | 0.00 |
| 6-10 | 0.33 (0.16-0.68) | 0.00 |
| 11-15 | 0.28 (0.13-0.59) | 0.00 |
| ≥16 | 1 [Reference] |  |
| Physical exercise |  |  |
| Yes | 1.90 (1.30-2.78) | 0.00 |
| No | 1 [Reference] |  |
| Chronic diseases |  |  |
| Yes | 0.62 (0.39-0.98) | 0.04 |
| No | 1 [Reference] |  |
| Smoking habit |  |  |
| Yes | 0.48 (0.29-0.82) | 0.00 |
| No | 1 [Reference] |  |
| Have any of your family members, friends, or colleagues been infected with the COVID-19? |  |  |
| Yes | 0.69 (0.49-0.99) | 0.04 |
| No | 1 [Reference] |  |
| Social support |  |  |
| Poor | 0.26 (0.17-0.39) | 0.00 |
| Moderate | 0.45 (0.30-0.66) | 0.00 |
| Strong | 1 [Reference] |  |
| ***Models for post-traumatic stress disorder symptoms*** |  |  |
| **Vaccinated teachers** |  |  |
| Residence |  |  |
| Urban | 1.50 (1.00-2.24) | 0.04 |
| Rural | 1 [Reference] |  |
| Do you have conducted an online class? |  |  |
| Yes | 2.24 (1.17-4.29) | 0.01 |
| No | 1 [Reference] |  |
| **Unvaccinated teachers** |  |  |
| Have any of your family members, friends, or colleagues died of the COVID-19? |  |  |
| Yes | 0.71 (0.26-0.90) | 0.01 |
| No | 1 [Reference] |  |
| ***Models for insomnia symptoms*** |  |  |
| **Vaccinated teachers** |  |  |
| Sex |  |  |
| Male | 1.55 (1.05-2.29) | 0.02 |
| Female | 1 [Reference] |  |
| Age, y |  |  |
| 24-35 | 0.68 (0.33-1.40) | 0.30 |
| 36-45 | 0.49 (0.24-1.00) | 0.05 |
| 46-55 | 0.69 (0.32-1.46) | 0.33 |
| ≥56 | 1 [Reference] |  |
| Social support |  |  |
| Poor | 0.36 (0.22-0.60) | 0.01 |
| Moderate | 0.40 (0.25-0.64) | 0.00 |
| Strong | 1 [Reference] |  |
| **Unvaccinated teachers** |  |  |
| Sex |  |  |
| Male | 1.45 (1.08-1.95) | 0.01 |
| Female | 1 [Reference] |  |
| Work experiences, y |  |  |
| <1 | 0.46 (0.20-1.06) | 0.06 |
| 1-5 | 0.35 (0.19-0.63) | 0.00 |
| 6-10 | 0.45 (0.24-0.83) | 0.01 |
| 11-15 | 0.41 (0.21-0.78) | 0.00 |
| ≥16 | 1 [Reference] |  |
| Physical exercise |  |  |
| Yes | 1.64 (1.15-2.34) | 0.00 |
| No | 1 [Reference] |  |
| Social support |  |  |
| Poor | 0.39 (0.27-0.57) | 0.00 |
| Moderate | 0.46 (0.32-0.66) | 0.00 |
| Strong | 1 [Reference] |  |
| ***Models for fear symptoms*** |  |  |
| **Vaccinated teachers** |  |  |
| Sex |  |  |
| Male | 2.47 (1.68-3.65) | 0.00 |
| Female | 1 [Reference] |  |
| Age, y |  |  |
| 24-35 | 0.20 (0.08-0.50) | 0.00 |
| 36-45 | 0.31 (0.13-0.77) | 0.01 |
| 46-55 | 0.77 (0.29-2.02) | 0.59 |
| ≥56 | 1 [Reference] |  |
| Education level |  |  |
| Masters or lower degree | 0.43 (0.23-0.79) | 0.00 |
| MPhil degree | 1.66 (0.44-6.19) | 0.44 |
| Doctoral degree | 0.93 (0.50-1.73) | 0.83 |
| Other | 1 [Reference] |  |
| Having children |  |  |
| Yes | 1.96 (1.34-2.87) | 0.00 |
| No | 1 [Reference] |  |
| Work experiences, y |  |  |
| <1 | 0.65 (0.07-5.75) | 0.70 |
| 1-5 | 0.19 (0.12-0.32) | 0.00 |
| 6-10 | 0.35 (0.20-0.61) | 0.00 |
| 11-15 | 0.33 (0.19-0.57) | 0.00 |
| ≥16 | 1 [Reference] |  |
| Have any of your family members, friends, or colleagues been infected with the COVID-19? |  |  |
| Yes | 1.74 (1.37-1.97) | 0.02 |
| No | 1 [Reference] |  |
| Have any of your family members, friends, or colleagues died of the COVID-19? |  |  |
| Yes | 0.58 (0.41-0.84) | 0.00 |
| No | 1 [Reference] |  |
| Social support |  |  |
| Poor | 0.46 (0.28-0.76) | 0.00 |
| Moderate | 0.55 (0.34-0.87) | 0.01 |
| Strong | 1 [Reference] |  |
| **Unvaccinated teachers** |  |  |
| Sex |  |  |
| Male | 1.91 (1.41-2.60) | 0.00 |
| Female | 1 [Reference] |  |
| Having children |  |  |
| Yes | 1.64 (1.22-2.21) | 0.00 |
| No | 1 [Reference] |  |
| Work experiences, y |  |  |
| <1 | 0.11 (0.04-0.30) | 0.00 |
| 1-5 | 0.19 (0.08-0.42) | 0.00 |
| 6-10 | 0.20 (0.08-0.46) | 0.00 |
| 11-15 | 0.32 (0.13-0.78) | 0.01 |
| ≥16 | 1 [Reference] |  |
| Physical exercise |  |  |
| Yes | 1.78 (1.21-2.61) | 0.00 |
| No | 1 [Reference] |  |
| Chronic diseases |  |  |
| Yes | 0.46 (0.29-0.72) | 0.00 |
| No | 1 [Reference] |  |
| Smoking habit |  |  |
| Yes | 0.53 (0.31-0.89) | 0.01 |
| No | 1 [Reference] |  |
| Personal COVID-19 infection |  |  |
| Yes | 1.74 (1.01-2.99) | 0.04 |
| No | 1 [Reference] |  |
| Have any of your family members, friends, or colleagues been infected with the COVID-19? |  |  |
| Yes | 1.24 (1.06-1.89) | 0.03 |
| No | 1 [Reference] |  |
| Have any of your family members, friends, or colleagues died of the COVID-19? |  |  |
| Yes | 0.64 (0.46-0.87) | 0.00 |
| No | 1 [Reference] |  |
| Social support |  |  |
| Poor | 0.34 (0.22-0.51) | 0.00 |
| Moderate | 0.38 (0.25-0.56) | 0.00 |
| Strong | 1 [Reference] |  |

Abbreviation: COR, Crude odds ratio; CI, confidence interval.
