# Supplementary material for: Mental health outcomes and associated factors among vaccinated and unvaccinated teachers against COVID-19 infection in Bangladesh
Source: Front Psychiatry. 2022 Aug 3;13:896419. doi: 10.3389/fpsyt.2022.896419 (PMC9382101; doi:10.3389/fpsyt.2022.896419)
Supplement: Supplementary file 2 [file Table_2.docx]

**Supplement Table S2.** Multivariate logistic regression analysis of factors associated with mental health outcomes among vaccinated and unvaccinated teachers against COVID-19 infection.

| **Variables** | **AOR (95% CI)** | ***p* value** |
| --- | --- | --- |
| ***Models for psychological distress symptoms*** |  |  |
| **Vaccinated teachers** |  |  |
| Work experiences, y |  |  |
| <1 | 1.59 (0.15-16.3) | 0.69 |
| 1-5 | 1.02 (0.42-2.43) | 0.96 |
| 6-10 | 0.69 (0.33-0.94) | 0.00 |
| 11-15 | 0.77 (0.41-1.44) | 0.42 |
| ≥16 | 1 [Reference] |  |
| Social support |  |  |
| Poor | 2.42 (1.55-3.80) | 0.00 |
| Moderate | 1.84 (1.25-2.70) | 0.00 |
| Strong | 1 [Reference] |  |
| **Unvaccinated teachers** |  |  |
| Age, y |  |  |
| 24-35 | 8.24 (1.57-43.0) | 0.00 |
| 36-45 | 7.80 (1.60-37.9) | 0.00 |
| 46-55 | 2.08 (0.46-9.45) | 0.34 |
| ≥56 | 1 [Reference] |  |
| Work experiences, y |  |  |
| <1 | 0.29 (0.08-0.95) | 0.03 |
| 1-5 | 0.36 (0.13-1.00) | 0.04 |
| 6-10 | 0.51 (0.19-1.36) | 0.18 |
| 11-15 | 0.41 (0.16-0.98) | 0.02 |
| ≥16 | 1 [Reference] |  |
| Physical exercise |  |  |
| Yes | 0.70 (0.49-1.00) | 0.03 |
| No | 1 [Reference] |  |
| Social support |  |  |
| Poor | 2.32 (1.58-3.43) | 0.00 |
| Moderate | 1.34 (0.95-1.90) | 0.09 |
| Strong | 1 [Reference] |  |
| ***Models for depression symptoms*** |  |  |
| **Vaccinated teachers** |  |  |
| Sex |  |  |
| Male | 1.78 (1.19-2.67) | 0.00 |
| Female | 1 [Reference] |  |
| Education level |  |  |
| Masters or lower degree | 1.31 (1.02-1.62) | 0.03 |
| MPhil degree | 1.23 (0.45-3.30) | 0.67 |
| Doctoral degree | 0.79 (0.57-0.93) | 0.02 |
| Other | 1 [Reference] |  |
| Having children |  |  |
| Yes | 1.39 (1.08-2.33) | 0.01 |
| No | 1 [Reference] |  |
| Work experiences, y |  |  |
| <1 | 0.52 (0.08-3.39) | 0.49 |
| 1-5 | 1.80 (1.34-2.86) | 0.01 |
| 6-10 | 0.80 (0.39-1.61) | 0.23 |
| 11-15 | 0.86 (0.47-1.58) | 0.63 |
| ≥16 | 1 [Reference] |  |
| Have any of your family members, friends, or colleagues been infected with the COVID-19? |  |  |
| Yes | 0.53 (0.31-0.91) | 0.02 |
| No | 1 [Reference] |  |
| Social support |  |  |
| Poor | 1.59 (1.37-2.92) | 0.02 |
| Moderate | 1.57 (1.22-2.85) | 0.00 |
| Strong | 1 [Reference] |  |
| **Unvaccinated teachers** |  |  |
| Sex |  |  |
| Male | 1.55 (1.11-2.15) | 0.00 |
| Female | 1 [Reference] |  |
| Education level |  |  |
| Masters or lower degree | 2.18 (0.95-4.99) | 0.06 |
| MPhil degree | 2.12 (0.76-5.88) | 0.14 |
| Doctoral degree | 2.29 (1.01-5.19) | 0.04 |
| Other | 1 [Reference] |  |
| Chronic diseases |  |  |
| Yes | 0.51 (0.32-0.83) | 0.00 |
| No | 1 [Reference] |  |
| Social support |  |  |
| Poor | 1.57 (1.17-2.83) | 0.00 |
| Moderate | 1.52 (1.09-2.81) | 0.00 |
| Strong | 1 [Reference] |  |
| ***Models for anxiety symptoms*** |  |  |
| **Vaccinated teachers** |  |  |
| Having children |  |  |
| Yes | 1.24 (1.02-2.13) | 0.02 |
| No | 1 [Reference] |  |
| Work experiences, y |  |  |
| <1 | 1.02 (0.13-7.54) | 0.98 |
| 1-5 | 1.68 (1.17-5.89) | 0.00 |
| 6-10 | 0.87 (0.42-1.81) | 0.71 |
| 11-15 | 1.05 (0.56-1.98) | 0.85 |
| ≥16 | 1 [Reference] |  |
| Chronic diseases |  |  |
| Yes | 0.64 (0.42-0.97) | 0.03 |
| No | 1 [Reference] |  |
| Smoking habit |  |  |
| Yes | 1.34 (1.12-2.14) | 0.00 |
| No | 1 [Reference] |  |
| Have any of your family members, friends, or colleagues died of the COVID-19? |  |  |
| Yes | 1.58 (1.27-2.84) | 0.00 |
| No | 1 [Reference] |  |
| Social support |  |  |
| Poor | 0.63 (0.26-0.99) | 0.00 |
| Moderate | 1.40 (1.11-1.82) | 0.00 |
| Strong | 1 [Reference] |  |
| **Unvaccinated teachers** |  |  |
| Sex |  |  |
| Male | 1.72 (1.23-2.40) | 0.00 |
| Female | 1 [Reference] |  |
| Physical exercise |  |  |
| Yes | 0.80 (0.22-0.91) | 0.00 |
| No | 1 [Reference] |  |
| Chronic diseases |  |  |
| Yes | 0.44 (0.27-0.73) | 0.00 |
| No | 1 [Reference] |  |
| Smoking habit |  |  |
| Yes | 1.35 (1.19-2.63) | 0.00 |
| No | 1 [Reference] |  |
| Social support |  |  |
| Poor | 1.36 (1.03-1.64) | 0.00 |
| Moderate | 1.57 (1.27-1.83) | 0.00 |
| Strong | 1 [Reference] |  |
| ***Models for stress symptoms*** |  |  |
| **Vaccinated teachers** |  |  |
| Education level |  |  |
| Masters or lower degree | 1.44 (1.21-3.12) | 0.03 |
| MPhil degree | 1.57 (0.36-6.68) | 0.54 |
| Doctoral degree | 1.96 (1.45-3.05) | 0.02 |
| Other | 1 [Reference] |  |
| Marital status |  |  |
| Single | 1.19 (0.22-6.38) | 0.83 |
| Married | 1.68 (1.37-7.65) | 0.00 |
| Divorced/separated/widowed | 1 [Reference] |  |
| Having children |  |  |
| Yes | 1.41 (1.18-2.26) | 0.02 |
| No | 1 [Reference] |  |
| Do you have conducted an online class? |  |  |
| Yes | 2.82 (1.26-6.28) | 0.01 |
| No | 1 [Reference] |  |
| Work experiences, y |  |  |
| <1 | 0.30 (0.03-2.43) | 0.26 |
| 1-5 | 1.89 (1.28-2.76) | 0.04 |
| 6-10 | 0.77 (0.30-1.98) | 0.60 |
| 11-15 | 0.96 (0.41-2.24) | 0.94 |
| ≥16 | 1 [Reference] |  |
| Social support |  |  |
| Poor | 0.77 (0.24-0.90) | 0.00 |
| Moderate | 0.97 (0.40-1.21) | 0.20 |
| Strong | 1 [Reference] |  |
| **Unvaccinated teachers** |  |  |
| Sex |  |  |
| Male | 1.63 (1.14-2.33) | 0.00 |
| Female | 1 [Reference] |  |
| Having children |  |  |
| Yes | 1.67 (1.09-2.55) | 0.01 |
| No | 1 [Reference] |  |
| Work experiences, y |  |  |
| <1 | 1.03 (0.25-4.18) | 0.95 |
| 1-5 | 1.39 (1.14-1.61) | 0.02 |
| 6-10 | 0.65 (0.21-2.05) | 0.46 |
| 11-15 | 0.49 (0.17-1.43) | 0.19 |
| ≥16 | 1 [Reference] |  |
| Physical exercise |  |  |
| Yes | 0.37 (0.11-0.88) | 0.04 |
| No | 1 [Reference] |  |
| Chronic diseases |  |  |
| Yes | 0.47 (0.28-0.79) | 0.00 |
| No | 1 [Reference] |  |
| Smoking habit |  |  |
| Yes | 1.41 (1.08-1.74) | 0.00 |
| No | 1 [Reference] |  |
| Have any of your family members, friends, or colleagues been infected with the COVID-19? |  |  |
| Yes | 0.65 (0.43-0.97) | 0.03 |
| No | 1 [Reference] |  |
| Social support |  |  |
| Poor | 1.69 (1.18-2.44) | 0.00 |
| Moderate | 1.49 (1.16-2.75) | 0.00 |
| Strong | 1 [Reference] |  |
| ***Models for post-traumatic stress disorder symptoms*** |  |  |
| **Vaccinated teachers** |  |  |
| Do you have conducted an online class? |  |  |
| Yes | 1.91 (1.22-3.89) | 0.03 |
| No | 1 [Reference] |  |
| Work experiences, y |  |  |
| <1 | 0.24 (0.03-1.57) | 0.13 |
| 1-5 | 0.61 (0.25-1.51) | 0.29 |
| 6-10 | 0.63 (0.29-1.34) | 0.23 |
| 11-15 | 0.48 (0.25-0.92) | 0.02 |
| ≥16 | 1 [Reference] |  |
| Have any of your family members, friends, or colleagues died of the COVID-19? |  |  |
| Yes | 1.29 (1.02-1.48) | 0.00 |
| No | 1 [Reference] |  |
| **Unvaccinated teachers** |  |  |
| Have any of your family members, friends, or colleagues died of the COVID-19? |  |  |
| Yes | 0.77 (0.23-0.90) | 0.00 |
| No | 1 [Reference] |  |
| ***Models for insomnia symptoms*** |  |  |
| **Vaccinated teachers** |  |  |
| Age, y |  |  |
| 24-35 | 0.63 (0.20-1.97) | 0.43 |
| 36-45 | 0.40 (0.17-0.97) | 0.04 |
| 46-55 | 0.67 (0.29-1.51) | 0.33 |
| ≥56 | 1 [Reference] |  |
| Social support |  |  |
| Poor | 0.71 (0.20-0.91) | 0.00 |
| Moderate | 0.77 (0.24-0.98) | 0.00 |
| Strong | 1 [Reference] |  |
| **Unvaccinated teachers** |  |  |
| Sex |  |  |
| Male | 1.38 (1.06-1.93) | 0.05 |
| Female | 1 [Reference] |  |
| Social support |  |  |
| Poor | 1.31 (1.07-1.61) | 0.00 |
| Moderate | 1.37 (1.01-1.68) | 0.00 |
| Strong | 1 [Reference] |  |
| ***Models for fear symptoms*** |  |  |
| **Vaccinated teachers** |  |  |
| Sex |  |  |
| Male | 2.99 (1.86-4.80) | 0.00 |
| Female | 1 [Reference] |  |
| Education level |  |  |
| Masters or lower degree | 1.50 (1.14-3.26) | 0.00 |
| MPhil degree | 5.05 (1.18-21.6) | 0.02 |
| Doctoral degree | 1.46 (0.74-2.86) | 0.26 |
| Other | 1 [Reference] |  |
| Having children |  |  |
| Yes | 1.49 (1.12-1.87) | 0.01 |
| No | 1 [Reference] |  |
| Work experiences, y |  |  |
| <1 | 0.91 (0.07-10.5) | 0.94 |
| 1-5 | 0.15 (0.05-0.43) | 0.07 |
| 6-10 | 0.25 (0.11-0.59) | 0.00 |
| 11-15 | 0.28 (0.13-0.60) | 0.00 |
| ≥16 | 1 [Reference] |  |
| Have any of your family members, friends, or colleagues died of the COVID-19? |  |  |
| Yes | 1.44 (1.05-1.88) | 0.00 |
| No | 1 [Reference] |  |
| **Unvaccinated teachers** |  |  |
| Sex |  |  |
| Male | 1.78 (1.24-2.56) | 0.00 |
| Female | 1 [Reference] |  |
| Having children |  |  |
| Yes | 0.71 (0.58-0.99) | 0.01 |
| No | 1 [Reference] |  |
| Chronic diseases |  |  |
| Yes | 0.32 (0.19-0.53) | 0.00 |
| No | 1 [Reference] |  |
| Smoking habit |  |  |
| Yes | 1.38 (1.07-2.70) | 0.00 |
| No | 1 [Reference] |  |
| Personal COVID-19 infection |  |  |
| Yes | 1.87 (1.04-3.35) | 0.03 |
| No | 1 [Reference] |  |
| Have any of your family members, friends, or colleagues died of the COVID-19? |  |  |
| Yes | 1.67 (1.14-1.96) | 0.02 |
| No | 1 [Reference] |  |
| Social support |  |  |
| Poor | 1.86 (1.08-2.58) | 0.00 |
| Moderate | 1.90 (1.12-2.57) | 0.00 |
| Strong | 1 [Reference] |  |

Abbreviation: AOR, Adjusted odds ratio; CI, confidence interval.
