# Supplementary figures and images for: Mental health outcomes and associated factors among vaccinated and unvaccinated teachers against COVID-19 infection in Bangladesh
Source: Front Psychiatry. 2022 Aug 3;13:896419. doi: 10.3389/fpsyt.2022.896419 (PMC9382101; doi:10.3389/fpsyt.2022.896419)

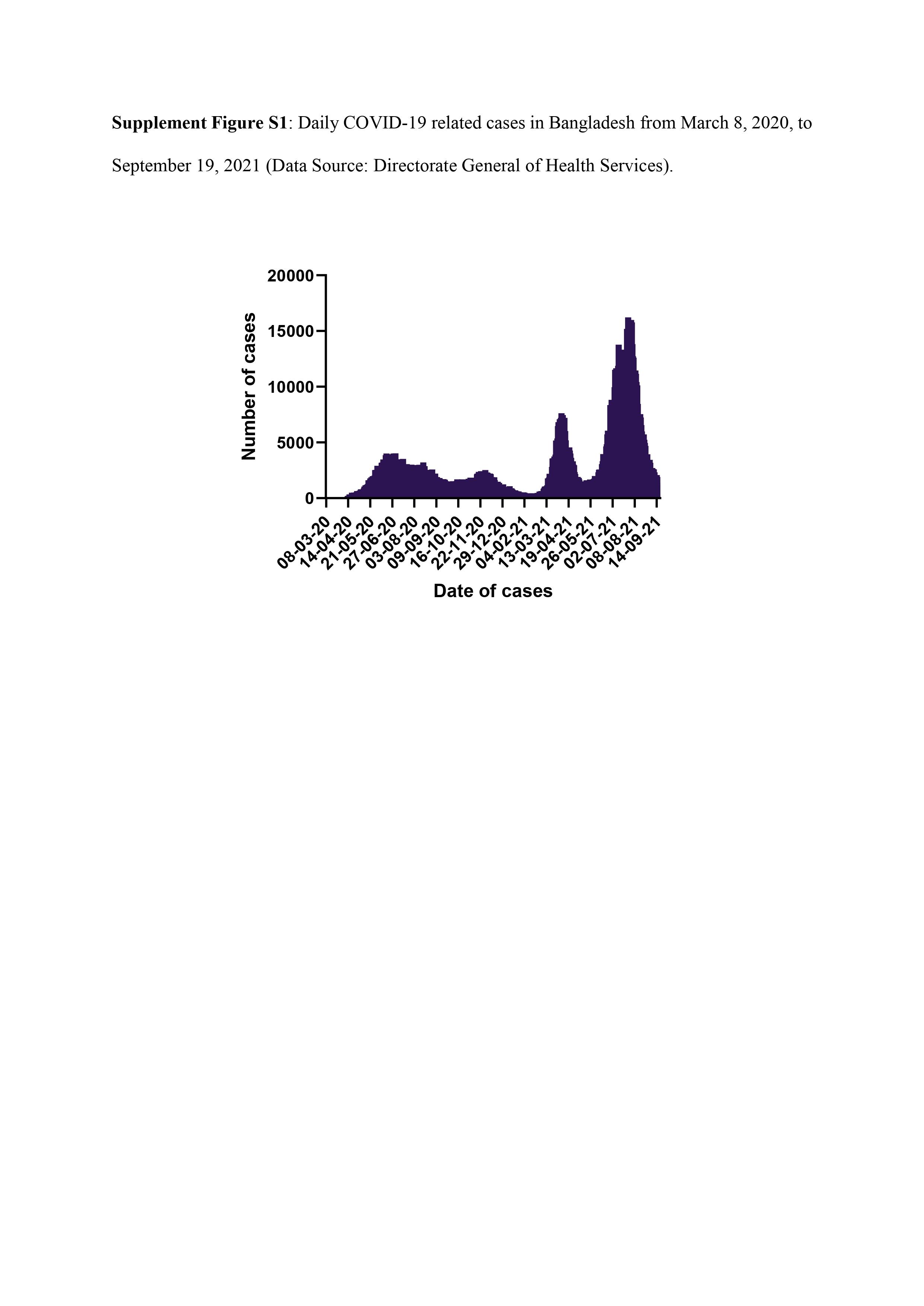

Supplement: Supplementary file 3 [file Image_1.jpg]

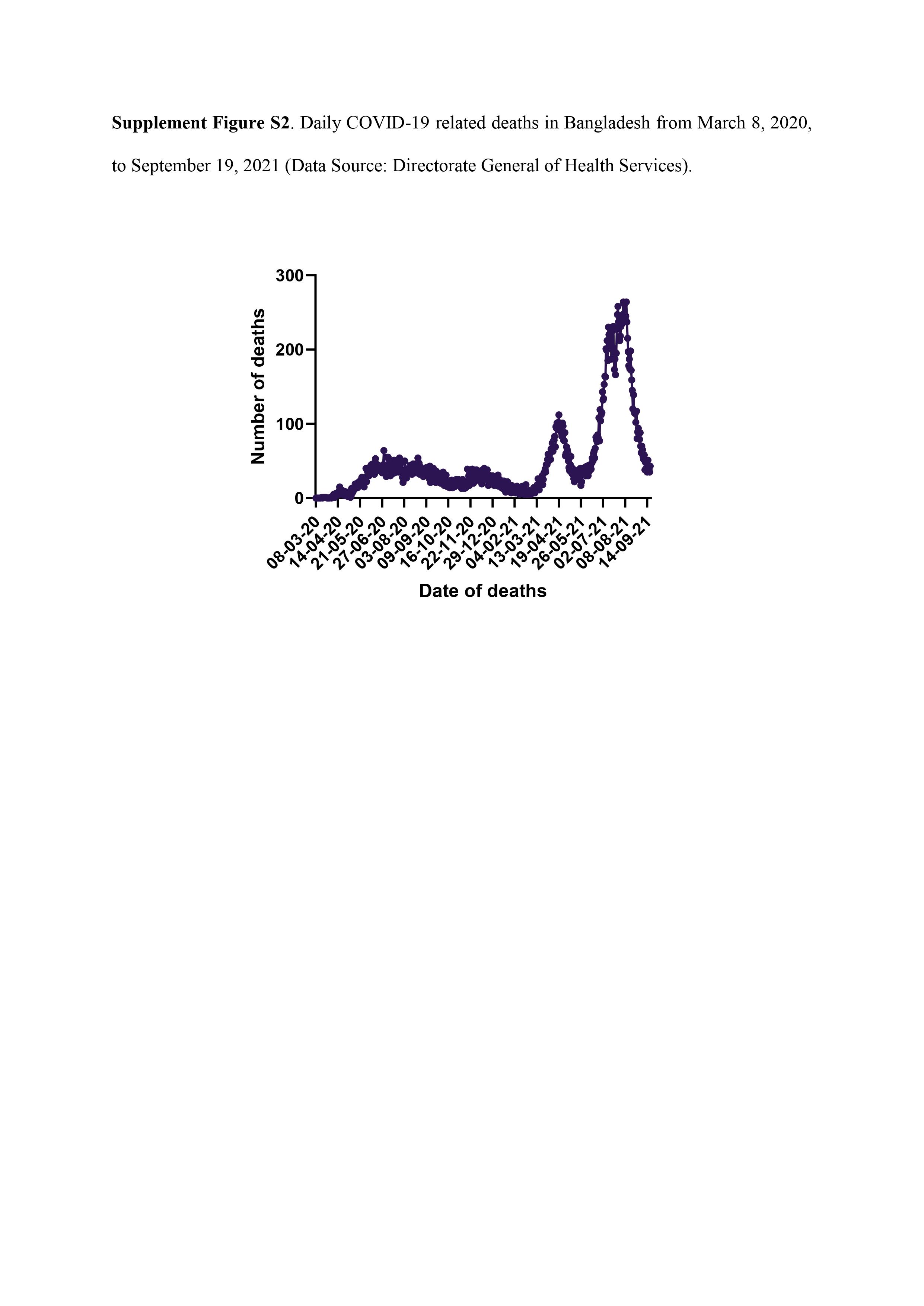

Supplement: Supplementary file 4 [file Image_2.jpg]

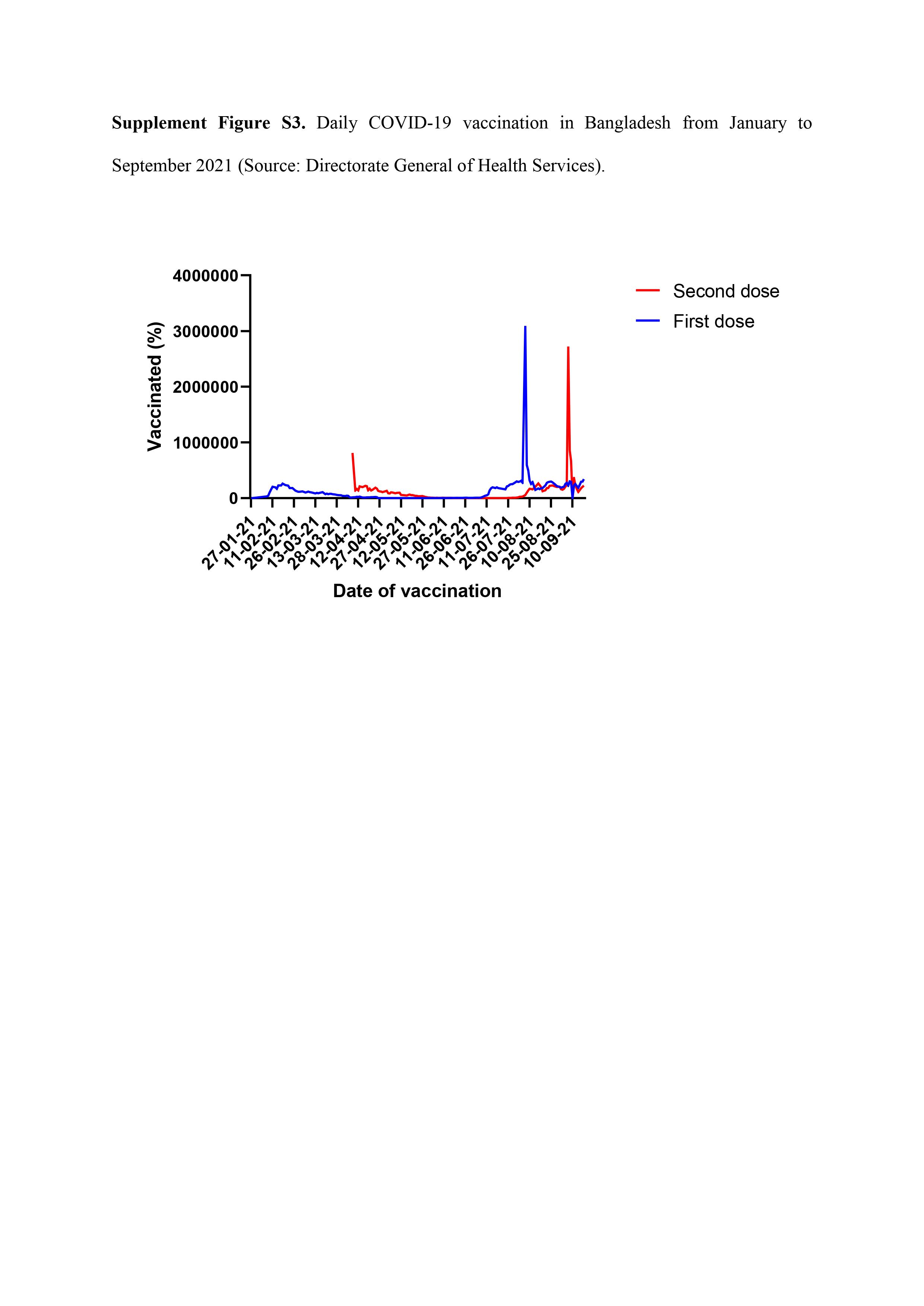

Supplement: Supplementary file 5 [file Image_3.jpg]
